# Spindle Assembly Checkpoint Competency Determines Sensitivity to KIF18A Inhibition in Small Cell Lung Cancer

**DOI:** 10.1158/2767-9764.CRC-26-0560

**Published:** 2026-09-09

**Authors:** Chiori Tabe, Rajesh Kumar, Yue Huang, Michael J. Kruhlak, Ajit Kumar Sharma, Roshan L. Shrestha, Anish Thomas

**Affiliations:** 1 Developmental Therapeutics Branch, Center for Cancer Research, National Cancer Institute, National Institutes of Health, Bethesda, Maryland.; 2 Laboratory of Cancer Biology and Genetics, Center for Cancer Research, National Cancer Institute, National Institutes of Health, Bethesda, Maryland.; 3 Division of Hematology and Oncology, Department of Medicine, Northwestern University Feinberg School of Medicine, Chicago, Illinois.

## Abstract

Small cell lung cancer (SCLC) is characterized by pervasive chromosomal instability (CIN) and remains largely refractory to targeted therapies. KIF18A, a motor protein that regulates chromosome alignment during mitosis, has emerged as a selective dependency in CIN-high tumors. Whether this dependency extends to SCLC, a prototypical CIN-high cancer, has not been established, and biomarkers predicting response to KIF18A inhibition, currently in clinical trials, are lacking. We integrated analyses of patient tumor datasets, neuroendocrine (NE) and non-NE SCLC cell lines, and functional perturbation models to define the determinants of response to KIF18A inhibition. CIN metrics, transcriptional programs, mitotic dynamics, and spindle assembly checkpoint (SAC) function were assessed using genomic profiling, live-cell imaging, genetic perturbation, and pharmacologic inhibition. *KIF18A* expression was elevated in SCLC tumors and correlated with CIN-associated transcriptional programs, proliferative markers, and NE status; however, these features did not predict sensitivity to KIF18A inhibition. Instead, response was determined by the functional integrity of the SAC. SAC-proficient SCLC cells underwent sustained mitotic arrest followed by apoptotic cell death upon KIF18A inhibition, whereas SAC-defective cells failed to maintain checkpoint activation and survived. Mechanistically, resistant cells exhibited impaired kinetochore recruitment of core SAC components, including MAD1L1 (MAD1) and BUB1B (BUBR1). Importantly, transient induction of acute CIN through TTK (MPS1) inhibition partially restored sensitivity to KIF18A inhibition in resistant models. This study provides the first mechanistic characterization of KIF18A dependency in SCLC, identifying SAC competency as the primary determinant of response. These findings establish a biologically informed framework for patient stratification and rational combination strategies.

**Significance:**

This study suggests that functional mitotic checkpoint activity may contribute to KIF18A inhibitor sensitivity in small cell lung cancer models. These findings provide a rationale for further evaluating mitotic checkpoint function as a candidate biomarker and for exploring KIF18A-targeted strategies in selected small cell lung cancer contexts.

## Introduction

Small cell lung cancer (SCLC) is a highly aggressive neuroendocrine (NE) carcinoma characterized by rapid disease progression, early metastatic dissemination, and poor prognosis (1). Although initially sensitive to chemotherapy, most patients relapse within months, and the 5-year survival rate remains below 7% (2). Genomic profiling has revealed near-universal inactivation of *TP53* and *RB1* and widespread genomic rearrangements, distinguishing SCLC from non–small cell lung cancer and highlighting the paucity of recurrent, targetable oncogenic drivers (3). Nevertheless, SCLC biology is influenced by additional oncogenic and signaling programs beyond *TP53/RB1* loss. In particular, MYC-family activation and NOTCH pathway alterations have been implicated in tumor heterogeneity, lineage plasticity, NE differentiation, and proliferative behavior in SCLC (4–7). As a result, therapeutic advances have been limited for decades, underscoring the urgent need to identify biologically stratified vulnerabilities that can be exploited for durable clinical benefit.

Chromosomal instability (CIN) is a pervasive feature of many cancers and has been implicated in driving malignant progression, intratumoral heterogeneity, and therapy resistance (8, 9). SCLC frequently exhibits high proliferative activity, extensive copy-number alterations, structural rearrangements, and replication stress (10–12), reflecting the marked genomic instability that characterizes this disease (2, 3, 13, 14). Importantly, chromosomally unstable cells often rely on specific mitotic regulators to maintain genome integrity and viability, revealing potential synthetic lethal vulnerabilities (15). Despite the extensive genomic instability that defines SCLC (3, 16), the extent to which established CIN-associated mitotic dependencies apply to SCLC and whether these dependencies are modified by SCLC subtype or driver-oncogene context remain incompletely defined.

SCLC exhibits marked transcriptional and phenotypic heterogeneity. Distinct molecular subtypes driven by ASCL1, NEUROD1, POU2F3, and YAP1 differ in NE differentiation, proliferative behavior, metabolic programs, and therapeutic response (17). Moreover, intratumoral heterogeneity and lineage plasticity enable dynamic transitions between states, particularly under treatment pressure, complicating patient stratification and biomarker development (4). MYC-family activity has also been associated with subtype evolution, NE differentiation, and NOTCH-related lineage programs in SCLC (6, 18). These observations raise the possibility that apparent associations between a therapeutic target and SCLC subtype may reflect underlying differences in proliferation, CIN-associated transcriptional programs, or driver oncogene state rather than direct subtype-specific regulation. As a result, vulnerabilities inferred from static genomic features may not uniformly translate across SCLC subtypes or cellular states, underscoring the need for functionally defined dependencies.

Faithful chromosome segregation during mitosis depends on the coordinated activity of microtubule–kinetochore–associated and mitotic checkpoint complex–associated proteins that function to monitor and maintain the integrity of the spindle assembly checkpoint (SAC). KIF18A, a member of the kinesin 8 family, is a molecular motor that regulates microtubule dynamics to promote accurate chromosome alignment and segregation (15, 19, 20). Loss or inhibition of KIF18A leads to persistent chromosome misalignment, activation of the SAC, prolonged mitotic arrest, and subsequent cell death (19, 21). This KIF18A-associated vulnerability has been demonstrated in multiple chromosomally unstable or aneuploid cancer contexts. Genetic studies showed that aneuploid cancer cells are vulnerable to perturbation of SAC-related mitotic programs and to KIF18A depletion and that CIN-positive breast and colorectal cancer cells specifically require KIF18A for proliferation (15, 22). More recent pharmacologic studies have extended these findings using selective KIF18A inhibitors in CIN-high tumor models, including high-grade serous ovarian cancer, triple-negative breast cancer, colorectal cancer, and broad cancer cell line panels (23–25). In addition, recent mechanistic work has shown that KIF18A inhibitor sensitivity is influenced by the balance between SAC signaling and anaphase-promoting complex/cyclosome (APC/C) activity (21, 23). This evidence provides the foundation for evaluating whether KIF18A–CIN/SAC vulnerability is operative in SCLC.

Pharmacologic targeting of KIF18A has recently become feasible. Selective KIF18A-directed compounds, including AM-series inhibitors and VLS-1272, have demonstrated preclinical efficacy in CIN-high tumor models by disrupting chromosome congression, altering KIF18A localization or motor activity, inducing mitotic accumulation, and promoting cell death (23–25). Moreover, KIF18A inhibitors have already entered early-phase clinical development, including VLS-1488 in advanced solid tumors, sovilnesib in high-grade serous ovarian cancer, and ATX-295 in advanced or metastatic solid tumors. The major translational question is how to define disease-specific biomarkers, resistance mechanisms, and rational therapeutic contexts for KIF18A inhibition. Given the paucity of targetable oncogenic drivers and the high degree of CIN, targeting KIF18A could offer a novel therapeutic approach to selectively eliminate vulnerable SCLC subpopulations. However, because KIF18A inhibitor sensitivity is not expected to be dictated by target expression alone, defining the contributions of SCLC subtype, driver oncogene context, CIN/proliferation state, and functional SAC response will be essential for patient selection and rational combination strategies.

Here, we investigate the role of KIF18A in SCLC, assess the sensitivity of molecularly distinct SCLC models to a selective KIF18A inhibitor, and evaluate the molecular and functional features associated with differential drug responses. Rather than proposing KIF18A as a newly discovered mitotic vulnerability in CIN-high cancers, we test whether the previously described KIF18A–CIN/SAC dependency extends to SCLC and whether SCLC-specific lineage state, driver oncogene context, *KIF18A* expression, RNA sequencing (RNA-seq)–derived CIN/proliferation programs, and SAC function help explain response heterogeneity. Where applicable, we further benchmarked these findings against additional CIN-positive non-SCLC models to contextualize the SCLC data within the broader KIF18A inhibitor literature. Our findings support KIF18A as a candidate mitotic vulnerability in a subset of SCLC models and identify SAC activity as a candidate functional determinant of KIF18A inhibitor response in this disease, consistent with and extending prior observations in other CIN-high cancer models.

## Materials and Methods

### Cell lines and culture

DMS 273, COLO 668, and COR-L88 cells were obtained from MilliporeSigma. All remaining commercially available cell lines were obtained from the American Type Culture Collection (ATCC). The patient-derived RA22#12 cell line was established from tumor tissue obtained at autopsy from a patient with metastatic SCLC. NCI-H841, NCI-H1048, DMS 53, NCI-H1092, NCI-H2171, and NCI-H2029 cells were cultured in Dulbecco’s Modified Eagle Medium/Nutrient Mixture F-12 (DMEM/F-12); DMS 273 cells were cultured in DMEM; and all other cell lines were cultured in Roswell Park Memorial Institute 1640 (RPMI 1640) medium. All culture media were supplemented with 10% fetal bovine serum (FBS). Cell lines obtained from ATCC were authenticated by ATCC using short tandem repeat (STR) profiling, whereas DMS 273, COLO 668, and COR-L88 cells were authenticated by the European Collection of Authenticated Cell Cultures (ECACC) using STR–PCR profiling. RA22#12 cells were routinely monitored for consistency in morphology and growth characteristics. No independent STR-based authentication was performed. All cell lines were maintained at 37°C in a humidified atmosphere containing 5% CO_2_. All cell lines were routinely tested for *Mycoplasma* contamination using the Mycoplasma Detection Kit (MycoStrip; InvivoGen, cat. #rep-mys-50). Cells were cultured for up to 20 passages after thawing and used in all experiments.

### Generation of cell lines expressing H2B-GFP and H2B-RFP

Lentiviruses were produced in HEK-293T cells by transient transfection with pCMV-VSV-G (8454, Addgene), pCMV-dR8.2 dvpr (8455, Addgene), and pHIV-H2B-eGFP (91776, Addgene) or pHIV-H2B-mRFP (18982, Addgene) using TurboFectin 8.0 (TF81001, OriGene). Viral supernatants were collected and used to infect target cells in the presence of polybrene (2 μg/mL). GFP- or red fluorescent protein (RFP)–positive populations were isolated by flow cytometry and confirmed by fluorescence microscopy.

### Inhibitor treatments

Cells were treated with the KIF18A inhibitor AM-9022 (synthesized by Amgen) at the indicated concentrations and timepoints. The TTK (MPS1) inhibitor, reversine (656820-32-5, MedChemExpress), was used at 0.2 μmol/L to induce CIN or at 0.05 μmol/L for 48 hours to reduce SAC activity. To activate the SAC, cells were treated overnight with 50 ng/mL nocodazole (HY-13520, MedChemExpress) or 10 μmol/L monastrol (S8439, Selleckchem) for 3 hours.

### Immunoblotting

Cells were lysed in RIPA buffer supplemented with protease and phosphatase inhibitor cocktails and phenylmethylsulfonylfluoride. Protein concentrations were determined using a Bio-Rad protein assay. Equal amounts of protein were resolved by SDS-PAGE, transferred to polyvinylidene difluoride membranes, and probed with primary antibodies against KIF18A (A301-080A, Bethyl Laboratories) and α-tubulin (T9026, MilliporeSigma) followed by horseradish peroxidase–conjugated secondary antibodies. Signals were detected by chemiluminescence, and band intensities were quantified using ImageJ software and normalized to α-tubulin.

### Immunostaining

Cells grown on glass coverslips were fixed with ice-cold methanol, washed with PBS, and blocked with 2% BSA in phosphate-buffered saline with Tween 20. Cells were incubated with primary antibodies against MAD1 (MABE867, Sigma-Aldrich), BUBR1 (EPR22544-48, Abcam), KIF18A (A301-080A, Bethyl Laboratories) and α-tubulin (T9026, MilliporeSigma) overnight at 4°C followed by fluorophore-conjugated secondary antibodies Alexa Fluor 488–conjugated anti–mouse IgG (A11029) and Alexa Fluor 568–conjugated anti–rabbit IgG (A11036; Invitrogen). Nuclei were counterstained with 4′,6-diamidino-2-phenylindole (DAPI), and coverslips were mounted using antifade mounting medium. Images were acquired by fluorescence microscopy.

### Microscopy and quantitative immunofluorescence image analysis

For MAD1 and BUBR1 signal intensity measurement, MAD1 and BUBR1 immunostained cells were imaged on the DeltaVision Core system (Applied Precision/GE HealthCare) consisting of an Olympus IX70 inverted microscope (Olympus America) with 100× numerical aperture (NA) 1.4 oil immersion objective and a CoolSNAP HQ 12-bit camera (Photometrics) controlled by SoftWoRx software. Z-stacks of at least 10 focal planes, each of 10 μm, were acquired. Signal intensity was measured using the plot profile tool in SoftWoRx. To calculate fluorescence intensities, boxes of 8 × 8 pixels were drawn on kinetochore regions, as ascertained by co-localized foci of MAD1 and BUBR1. For background, four boxes of 8 × 8 pixels were drawn at four random areas within the cytoplasm in the same cell. The maximum intensity values from all drawn areas were obtained using the data inspector tool in SoftWoRx. Final fluorescence intensity for each protein was calculated by subtracting the average background intensity. For MAD1 signal intensity analysis in interphase cells, signal intensities were measured at five different spots in the nuclear membrane in a cell using point tool in Fiji. Average intensity from five spots was calculated to obtain average MAD1 nuclear membrane signal intensities in a cell.

### Time-lapse imaging

For time-lapse imaging, cells were seeded in μ-Slide 8-well coverglass bottom chambers (ibidi) and treated with AM-9022 and/or reversin as the experimental regime. Live-cell imaging was performed using a Nikon SoRa spinning disk confocal microscope equipped with a 40x plan-apochromat objective lens (NA, 0.95), Fusion BT scientific complementary metal-oxide-semiconductor camera (Hamamatsu Photonics K.K.), and stage top incubator (Tokai Hit USA, Inc.) to maintain humidity, a 37°C temperature, and 5% CO_2_. Z-stacked (0.16 μm X–Y pixel size and 1.5 μm z-step size) images were acquired from multiple positions in each well every 4 minutes for 48 hours. Image analysis were performed using NIS-Elements software (v. 5.42; Nikon Instruments, Inc.).

### Cell viability assay (ATPlite)

Cell viability was assessed using the ATPlite luminescence assay (PerkinElmer) according to the manufacturer’s instructions. Cells were treated with serial dilutions of AM-9022 for 72 hours, and luminescence was measured using a microplate reader. IC_50_ values were calculated by nonlinear regression using GraphPad Prism.

### Cell viability assay (MTS)

Cell viability was also determined using the 3-(4,5-dimethylthiazol-2-yl)-5-(3-carboxymethoxyphenyl)-2-(4-sulfophenyl)-2H-tetrazolium (MTS) colorimetric assay (APExBIO). Following 72 hours of treatment with serial dilutions of AM-9022, absorbance at 490 nm was measured and IC_50_ values were calculated using GraphPad Prism.

### Cell growth assay

Cell proliferation was monitored over 72 hours following AM-9022 treatment using the Incucyte live-cell imaging system (Sartorius). Phase-contrast images were acquired every 6 hours, and confluence was quantified automatically using Incucyte software and plotted as a function of time.

### Doubling time and proliferation rate

Cell doubling times were measured following the manufacturer’s instructions (Cellecta). Viable cells were counted using ViaStain AOPI Staining Solution (Revvity), replated at a defined density (Xb), and recounted after a defined interval (Xe). Doubling time (Td) was calculated as Td = [T × ln(2)]/ln(Xe/Xb).

### Short Hairpin RNA–mediated knockdown

*KIF18A* knockdown was achieved using lentiviral short hairpin RNA (shRNA) vectors (SVSHU6T16-L, Cellecta). Cells were transduced according to the manufacturer’s instructions, and stable knockdown cell lines were established by selection for RFP expression and puromycin resistance. shRNA expression was induced with doxycycline (8 μg/mL), and knockdown efficiency was confirmed by immunoblotting. shRNA sequences are listed in Supplementary Table S1.

### Bioinformatics and transcriptomic analysis

Public SCLC cell line expression data were obtained from the Cancer Cell Line Encyclopedia and SCLC CellMinerCDB. For RNA-seq generated in this study, total RNA was isolated from SCLC cell lines using the RNeasy kit (Qiagen, 74134) per the manufacturer’s protocol. RNA integrity was assessed on an Agilent 4150 TapeStation, and samples meeting quality thresholds were submitted to Novogene for library preparation and sequencing. Poly(A)-selected libraries were constructed and sequenced on an Illumina NovaSeq platform (paired-end 150 bp), yielding ∼6 Gb of raw data per sample. Raw FASTQ files underwent quality control using FastQC, followed by adapter trimming and quality filtering using Trimmomatic. Filtered reads were aligned to the human reference genome using STAR (v2.7.11b). Gene-level counts were quantified with featureCounts (Rsubread). Downstream analyses were performed in R (v4.2.3). Differential expression between AM-9022–sensitive and –resistant cell lines was performed using DESeq2, and genes with |log_2_ fold change| ≥ 1.5 and nominal *P* < 0.05 were carried forward for pathway analyses (unless otherwise specified). Gene set enrichment analysis (GSEA) was performed using the Broad Institute Molecular Signatures Database gene sets. Where indicated, single-sample GSEA was used to score predefined signatures [e.g., centromere and kinetochore gene expression score (CES)] across cell lines.

### NE score and NE status assignment

NE status was assigned using the previously described Zhang NE score (26), an expression-based 50-gene signature consisting of 25 NE-associated and 25 non–NE-associated genes. NE scores were calculated from the corresponding RNA-seq dataset used for each cell line. The score ranges from −1 to +1, with positive values indicating an NE-like state and negative values indicating a non–NE-like state. NE status was therefore defined by this expression-based score and not by driver oncogene alterations.

### Statistics

Statistical analyses of experimental data were performed using GraphPad Prism (version 10). Statistical tests were selected based on data distribution and sample size. For multiple-group comparisons, Kruskal–Wallis with the Dunn test or one-way ANOVA with the Tukey test was used as appropriate. For repeated measures, Friedman tests were applied. Two-way ANOVA was used for experiments involving two independent variables. Pairwise comparisons were performed using a two-sided Wilcoxon rank-sum or Welch *t* test. Data are presented as mean ± SD unless otherwise indicated. A *P* value < 0.05 was considered statistically significant.

## Results

### *KIF18A* expression is associated with CIN and NE features in SCLC

To investigate the genomic instability landscape of SCLC, we first compared indices that capture CIN and aneuploidy, including the fraction genome altered (FGA; ref. 27), CES (28), and the CIN70 (29) scores, across lung cancer histologic subtypes. Lung adenocarcinoma and lung squamous cell carcinoma (LUSC) primary tumor datasets were obtained from The Cancer Genome Atlas (30), and SCLC tumors were derived from the George and colleagues cohort (3).

**Figure 1.**
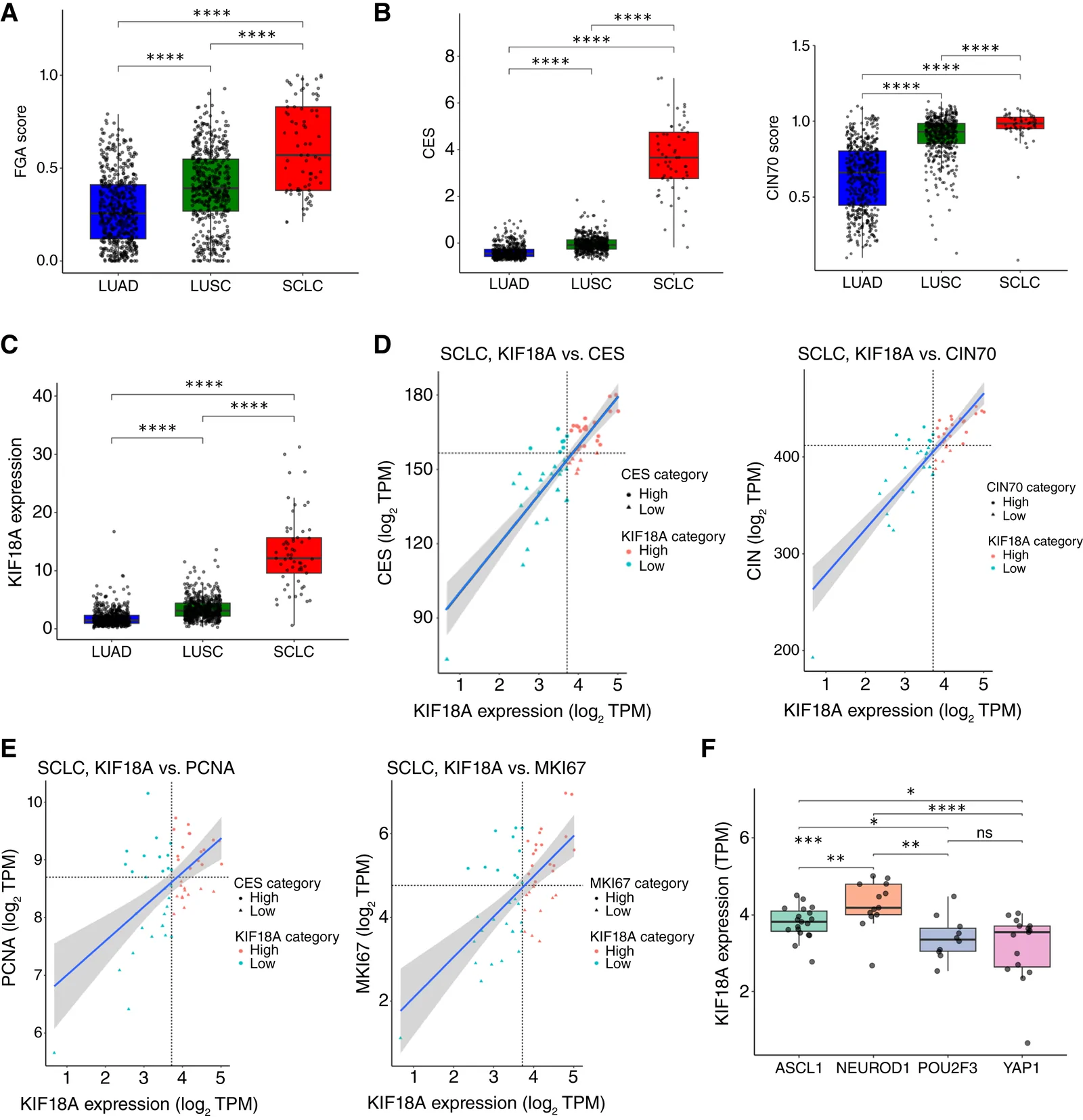
Association of KIF18A expression with CIN in SCLC. **A,** Box plot showing the FGA scores across different lung cancer subtypes, including lung adenocarcinoma (LUAD, *n* = 518) and LUSC (*n* = 500) from The Cancer Genome Atlas (TCGA) database (30), and SCLC (*n* = 58) from George and colleagues (3). **B,** Box plots showing CES (left; ref. 28) and CIN70 gene signature (right; ref. 29) using TCGA (30) and George and colleagues datasets. **C,** Box plot showing KIF18A transcript levels across lung cancer subtypes in TCGA and George and colleagues datasets. **D,** Scatter plots showing the relationship between KIF18A transcript levels and CES (left) and CIN70 (right), across SCLC tumors from the George and colleagues dataset. **E,** Scatter plots showing correlations between KIF18A expression and proliferation markers PCNA (left) and MKI67 (right) in the George and colleagues dataset. **F,** Box plots showing KIF18A transcript levels across molecular subtypes of SCLC (ASCL1, NEUROD1, POU2F3, and YAP1) in the George and colleagues dataset. *P* values in **A–C** and **F** were calculated using two-sided Wilcoxon tests. Correlations in **D** and **E** were assessed using Spearman rank correlation. ns, not statistically significant; *, *P* < 0.05; **, *P* < 0.01; ***, *P* < 0.001; ****, *P* < 0.0001. TPM, transcripts per million.

SCLC tumors exhibited significantly higher FGA score compared with lung adenocarcinoma and LUSC (Fig. 1A). Consistently, SCLC tumors exhibited pronounced CIN, as evidenced by significantly increased CES and CIN70 scores, relative to lung adenocarcinoma and LUSC cohorts (Fig. 1B). Taken these data together, we conclude that SCLC subtype of lung cancer is genomically and chromosomally instable compared with lung adenocarcinoma and LUSC.

Given the pronounced CIN observed in SCLC, we next asked whether expression of *KIF18A*, which encodes a mitotic motor protein previously implicated as a selective dependency in genomically unstable cancers (22), was elevated in SCLC. *KIF18A* expression indeed was significantly upregulated in SCLC tumors compared with lung adenocarcinoma and LUSC (Fig. 1C). Because the SCLC, lung adenocarcinoma, and LUSC datasets were derived from different sources, we next assessed whether the higher *KIF18A* expression in SCLC could be explained by dataset-level effects. Rather than applying conventional batch correction such as Combating Batch Effects When Combining Batches, given that tumor type and dataset source were fully confounded, we evaluated global expression differences using sample-wide and housekeeping gene expression metrics (Supplementary Fig. S1A–S1D). *KIF18A* expression remained significantly higher in SCLC after adjustment for these measures (Supplementary Fig. S1E). In addition, elevated *KIF18A* expression and high CESs were observed in independent SCLC cohorts, including the Liu and colleagues (31), Nabet and colleagues (32), and Park and colleagues (33) datasets, supporting the conclusion that these features are recurrent in SCLC rather than artifacts of a single dataset.

Next, we examined whether *KIF18A* expression correlated with CIN-related signatures.

Within SCLC, *KIF18A* expression strongly correlated with CES (r = 0.83, *P* < 0.0001) and CIN70 (r = 0.85, *P* < 0.0001) scores, consistent with *KIF18A* expression tracking with CIN-associated transcriptional signatures (Fig. 1D). We next investigated the relationship between *KIF18A* expression and proliferative capacity in SCLC. *KIF18A* expression also correlated positively with proliferation markers *PCNA* (r = 0.55, *P* < 0.0001) and *MKI67* (r = 0.62, *P* < 0.0001; Fig. 1E). Correlations between *KIF18A* expression and CIN, and proliferation were consistent in other datasets (Supplementary Fig. S1F). These data indicate that *KIF18A* expression is associated with cell-cycle/proliferation-related features in SCLC. Finally, we examined *KIF18A* expression across SCLC molecular subtypes as an additional descriptive analysis. *KIF18A* expression differed among subtypes, with relatively higher levels in ASCL1-driven and NEUROD1-driven tumors and lower levels in POU2F3-driven and YAP1-driven tumors (Fig. 1F). Because SCLC molecular subtypes may overlap with CIN-related and proliferative states, we compared CESs and *MKI67* expression across subtypes. Neither parameter was significantly enriched in ASCL1-driven or NEUROD1-driven tumors relative to POU2F3-driven or YAP1-driven tumors (Supplementary Fig. S1G and S1H). These findings suggest that the elevated *KIF18A* expression observed in NE subtypes is not explained by higher CIN or proliferation and may also reflect subtype-associated biology.

Together, these data show that *KIF18A* expression is elevated in SCLC relative to lung adenocarcinoma and LUSC and correlates with CIN- and proliferation-associated markers within datasets of patients with SCLC. These correlative findings provide a rationale for evaluating *KIF18A* expression and dependence on KIF18A across SCLC models.

### SCLC cell lines exhibit heterogeneous *KIF18A* expression independent of drug sensitivity

**Figure 2.**
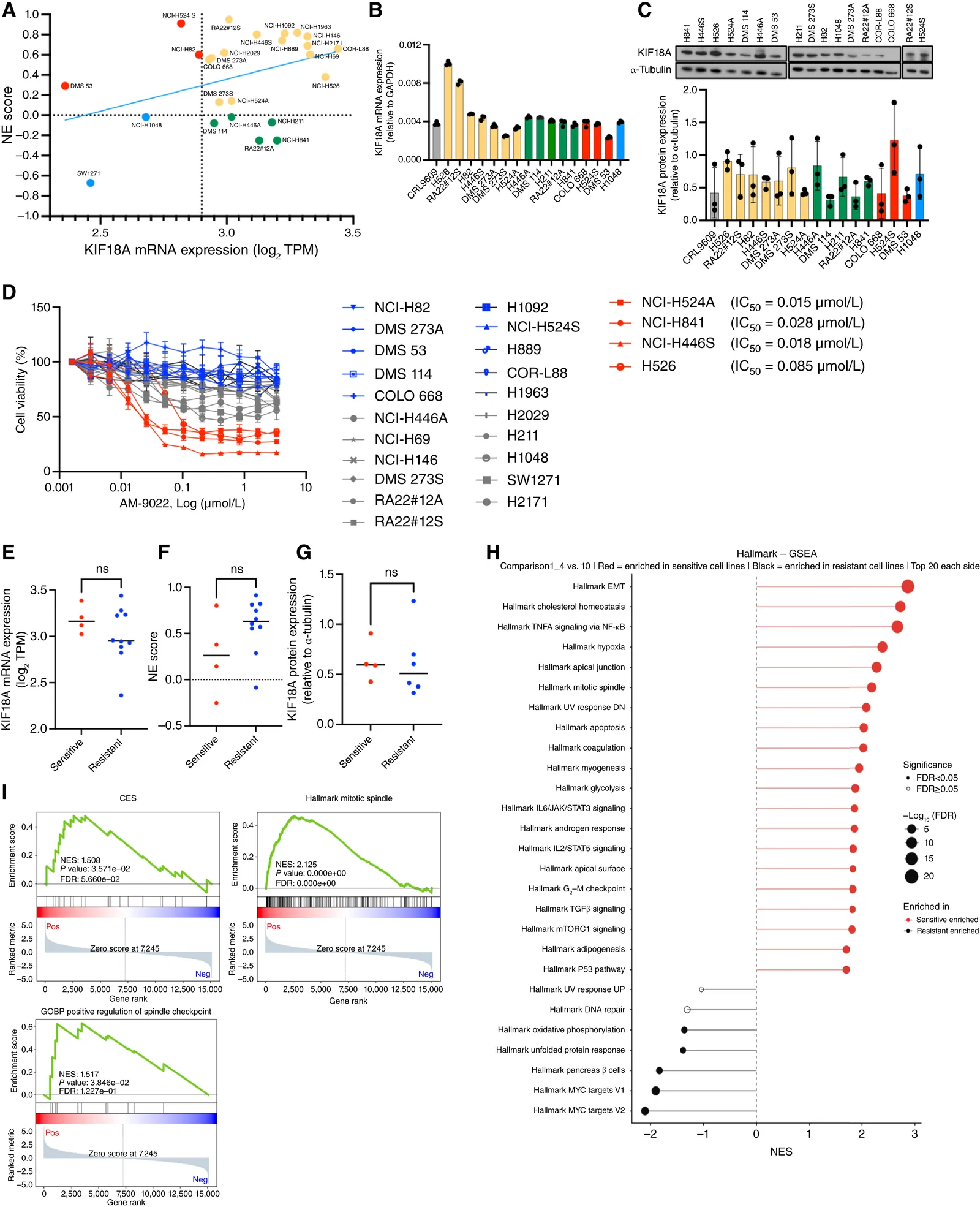
Molecular features associated with sensitivity to KIF18A inhibition in SCLC. **A,** Scatter plot showing the KIF18A mRNA expression and NE status across SCLC cell lines. NE scores were calculated according to Zhang and colleagues (26) using a 50-gene NE signature. Each datapoint represents an individual SCLC cell line. **B,** Bar graph showing relative KIF18A mRNA levels across SCLC cell lines as measured by quantitative RT-PCR. Expression was normalized to GAPDH. Data represent mean ± SD from three independent biological replicates. **C,** Western blot analysis of KIF18A protein levels across a panel of SCLC cell lines. α-Tubulin was used as the loading control. Bar graph showing relative KIF18A protein levels across SCLC cell lines as measured by immunoblotting. Protein expression was normalized to α-tubulin. Data represent mean ± SD from three independent biological replicates. **D,** Dose–response curves showing the sensitivity of SCLC cell lines to a KIF18A inhibitor, generated using technical replicates. Cell lines were grouped by drug response at the highest tested concentration: sensitive if viability was <50%, resistant if viability was ≥85% (Imax_obs < 15%), and partial/excluded if viability remained ≥50% despite measurable inhibition (Imax_obs ≥ 10%–15%). **E,** Dot plots showing KIF18A transcript levels for individual SCLC cell lines classified as KIF18A inhibitor sensitive or resistant. **F,** Dot plots showing NE scores for individual SCLC cell lines classified as KIF18A inhibitor sensitive or resistant. **G,** Dot plots showing KIF18A protein levels for individual SCLC cell lines classified as KIF18A inhibitor sensitive or resistant. **H,** Summary of GSEA comparing transcriptomic profiles of AM-9022–sensitive (*n* = 4) vs. –resistant (*n* = 10) SCLC cell lines. Gene sets with positive normalized enrichment scores (NES; red) are enriched in AM-9022–sensitive SCLC cell lines, whereas gene sets with negative NES (black) are enriched in resistant cell lines and depleted in sensitive cell lines. **I,** GSEA enrichment score plots showing enrichment of CIN (CES), mitotic spindle, and spindle checkpoint in AM-9022–sensitive SCLC cell lines relative to resistant cell lines. Statistical significance was determined using the Mann–Whitney U test. DN, downregulated; Imax_obs, observed maximum inhibition; Neg, negative; ns, not statistically significant; Pos, positive.

*KIF18A* mRNA expression levels varied widely among SCLC cell lines (34). Consistent with the trend observation in tumor data (Fig. 1C), *KIF18A* expression showed a weak positive trend (r = 0.39; *P* = 0.053; Fig. 2A) with the previously described Zhang NE score (26), an expression-based metric in which positive values indicate an NE-like state and negative values indicate a non–NE-like state. To further characterize *KIF18A* expression in our SCLC cell line panel, we experimentally examined *KIF18A* mRNA and KIF18A protein levels. Quantitative PCR confirmed heterogeneous *KIF18A* mRNA expression across representative SCLC cell lines (Fig. 2B). Western blotting likewise revealed variable protein expression across the panel (Fig. 2C). Notably, KIF18A protein levels did not simply reflect mRNA abundance, suggesting that transcript levels do not necessarily predict protein expression in these SCLC cell lines.

As KIF18A inhibitor has been reported to be lethal to chromosomally unstable cells (22, 24, 35), next we tested if KIF18A inhibitor was uniformly lethal across SCLC cell lines with varying *KIF18A* mRNA and KIF18A protein expression and KIF18A protein expr. We analyzed a panel of 25 SCLC cell lines spanning a broad range of molecular and phenotypic features, including NE, non-NE, adherent, or suspension growth patterns (Supplementary Table S2). Cells were treated with AM-9022, a selective ATP-competitive inhibitor of KIF18A that blocks its motor activity and impairs chromosome alignment during mitosis (24). Importantly, this mechanism is directly relevant to clinically advanced KIF18A inhibitors, including VLS-1488 and sovilnesib, which are currently undergoing phase I clinical evaluation in patients with solid tumors (ClinicalTrials.gov: NCT05902988 and NCT06084416; refs. 36, 37).

Dose–response analyses following 72-hour treatment revealed marked heterogeneity in sensitivity. Four cell lines (H841, H524A, H446S, and H526) exhibited low IC_50_ values and were classified as sensitive, whereas the majority of lines were resistant even at higher drug concentrations (Fig. 2D; Supplementary Fig. S2A). Importantly, *KIF18A* mRNA expression, KIF18A protein expression, and NE score did not differ significantly between sensitive and resistant cell lines (Fig. 2E–G). Consistent with this, neither *KIF18A* mRNA expression nor NE score showed a significant correlation with normalized AUC (KIF18A mRNA: r = −0.09; *P* = 0.66 and NE score: r = 0.14; *P* = 0.49; Supplementary Fig. S2B and S2C). Similarly, KIF18A protein levels did not correlate significantly with normalized AUC (r = −0.07; *P* = 0.80; Supplementary Fig. S2D). Together, these results indicate that although *KIF18A* mRNA expression seems to be associated with NE status in SCLC cell lines (Figs. 1F and 2A), neither NE status nor baseline *KIF18A* mRNA or KIF18A protein expression predicts sensitivity to KIF18A inhibition. In addition, *KIF18A* mRNA and KIF18A protein levels were not consistently concordant across the cell-line panel (Fig. 2B and C).

Given the essential role of KIF18A motor activity in chromosome alignment and mitotic progression, we hypothesized that sensitivity to KIF18A inhibition might reflect differences in cell-cycle kinetics that limit the accumulation of mitotic defects within the treatment window. To test this, we compared doubling time and proliferation rates between sensitive and resistant cell lines (Supplementary Table S3). No significant association was observed between doubling time and drug sensitivity (r = 0.37; *P* = 0.45; Supplementary Fig. S2E; Supplementary Table S3), suggesting that factors beyond proliferation rate or *KIF18A* mRNA expression, or KIF18A protein abund govern KIF18A dependency in SCLC.

To investigate the transcriptional features associated with the observed heterogeneity in response to KIF18A inhibition, we performed RNA-seq of SCLC cell lines stratified by their response to AM-9022. For this analysis, cell lines with <50% viability at the highest tested AM-9022 concentration were classified as sensitive, whereas those with ≥85% viability were classified as resistant; cell lines with partial or intermediate responses were excluded from this binary comparison. Using these criteria, four cell lines were classified as sensitive and 10 cell lines as resistant.

Pathway enrichment analysis revealed that the mitotic spindle regulation was one of the significantly enriched hallmarks in sensitive cell lines compared with resistant counterparts (Fig. 2H; Supplementary Fig. S2F). Additional pathway-level differences were also observed, including enrichment of epithelial–mesenchymal transition (EMT)–related gene sets in sensitive cell lines and enrichment of MYC target gene sets in resistant cell lines (Fig. 2H). A complementary pathway-level analysis showed enrichment of genes involved in mitotic spindle organization and SAC regulation in the sensitive group, with CIN-associated signatures, including CES, among the top-ranked pathways (Fig. 2I).

Collectively, these analyses indicate that KIF18A inhibitor sensitivity is accompanied by transcriptional differences in mitotic spindle organization, checkpoint-related cell-cycle processes, and stress response–related programs. The enrichment of SAC-associated gene sets in sensitive cell lines prompted further functional interrogation of mitotic checkpoint activity as a potential determinant of AM-9022 response.

### KIF18A inhibition selectively suppresses proliferation through defects in mitotic progression in sensitive SCLC cells

To functionally characterize the cellular response to KIF18A inhibition, we treated the AM-9022–sensitive H841 and resistant DMS 53 SCLC cell lines with increasing concentrations of AM-9022 and assessed their proliferation, cell viability, and mitotic dynamics across multiple timepoints.

**Figure 3.**
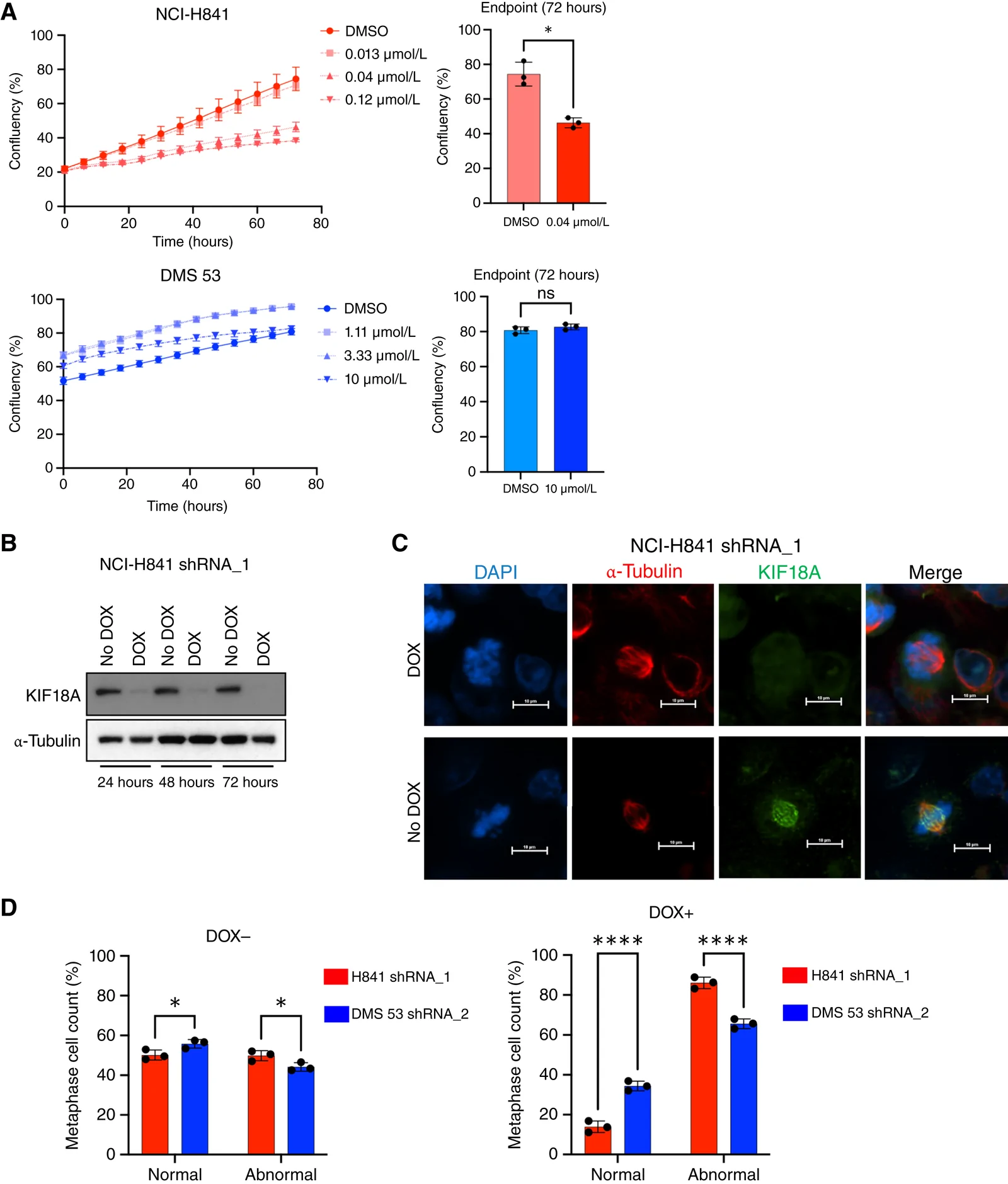
KIF18A inhibition selectively impairs proliferation and mitotic spindle integrity in sensitive SCLC cells. **A,** Incucyte live-cell imaging analysis for cell proliferation in NCI-H841 (top) and DMS 53 (bottom) SCLC cell lines treated with different concentrations of AM-9022 for multiple timepoints. Dot plots displaying endpoint confluency at 72 hours in NCI-H841 (top) and DMS 53 (bottom) cells treated with indicated concentrations of AM-9022. *P* values were calculated using the Welch test. ns, not statistically significant; *, *P* < 0.05. **B,** Immunoblot analysis of KIF18A protein levels following doxycycline (DOX)–induced shRNA expression in NCI-H841 cells. Cells were cultured in the absence or presence of DOX (8 μg/mL) for the indicated times (24, 48, and 72 hours). α-Tubulin was used as the loading control. **C,** Representative IF images of mitotic NCI-H841 cells following DOX-induced KIF18A knockdown using shRNA_1. Cells were immunostained with α-tubulin (red) and KIF18A (green). DNA was stained with DAPI (blue). Scale bars, 10 μm. **D,** Quantification of metaphase cells with normal or abnormal spindle organization in NCI-H841 and DMS 53 cells cultured in the absence of doxycycline (DOX−) or in the presence of doxycycline (DOX+) to induce KIF18A knockdown. Data from both cell lines were pooled for each doxycycline condition. For each biological replicate in each cell line and condition, 100 metaphase cells were scored and classified as normal or abnormal. Percentage of cells in each category. Bars indicate the mean, and dots represent individual biological replicates from each cell line (*n* = 6 per condition; 3 biological replicates per cell line). *, *P* < 0.05, ****, *P* < 0.0001.

Incucyte-based proliferation assays revealed a pronounced, dose-dependent suppression of proliferation in H841 cells, whereas DMS 53 cells exhibited only marginal growth inhibition even at the highest tested concentrations (Fig. 3A). At 72 hours, treatment with 0.04 μmol/L AM-9022 reduced confluence in H841 cells from 74% to 46% (∼38% inhibition; *P* = 0.01), with no effect in DMS 53 cells (*P* = 0.26). This differential response was corroborated by MTS cell viability assays, which showed a marked decrease in cell viability in H841 cells, with an IC_50_ of 0.032 μmol/L, in contrast to the largely unchanged viability profile observed in DMS 53 cells (Supplementary Fig. S3A). Collectively, these findings indicate that AM-9022 selectively impairs proliferative capacity in sensitive SCLC cells.

To delineate the mechanistic basis of this differential sensitivity, we examined mitotic progression following KIF18A inhibition, building on prior reports of spindle and chromosome congression defects upon KIF18A loss (19). The mitotic index was quantified in two KIF18A sensitive and two KIF18A resistant cell lines following treatment with AM-9022 for 72 hours. KIF18A inhibition resulted in a markedly increased mitotic index in sensitive cell lines, indicative of mitotic arrest (Supplementary Fig. S3B). In contrast, resistant cell lines exhibited a low mitotic index upon KIF18A inhibition (Supplementary Fig. S3B). These findings indicate that resistant cells fail to undergo mitotic arrest in response to KIF18A inhibition.

To determine whether these mitotic defects reflect an on-target requirement for KIF18A, we next examined the consequences of *KIF18A* knockdown. We generated H841 and DMS 53 cells harboring a doxycycline-inducible KIF18A knockdown and optimized the concentration and duration of doxycycline treatment, identifying 8 μg/mL as the condition that achieved efficient depletion (Supplementary Fig. S3C and S3D). Robust knockdown of KIF18A was observed following doxycycline treatment, as confirmed by immunoblotting (Fig. 3B) and immunofluorescence (IF) analyses (Fig. 3C). Although the proportions of normal and abnormal metaphases were comparable in both cell lines in the presence of KIF18A [Fig. 3D (DOX−)], genetic depletion of KIF18A resulted in a significant increase in the proportion of abnormal metaphase cells in both cell lines [Fig. 3D (DOX+)]. Notably, KIF18A depletion led to a more modest increase in abnormal metaphase cells in the sensitive cell line [H841; Fig. 3D (DOX+)]. Collectively, these results demonstrate that KIF18A is required for proper metaphase chromosome alignment and mitotic progression in sensitive SCLC cells.

### KIF18A inhibition induces SAC-dependent mitotic arrest and cell death in the sensitive SCLC cell line

**Figure 4.**
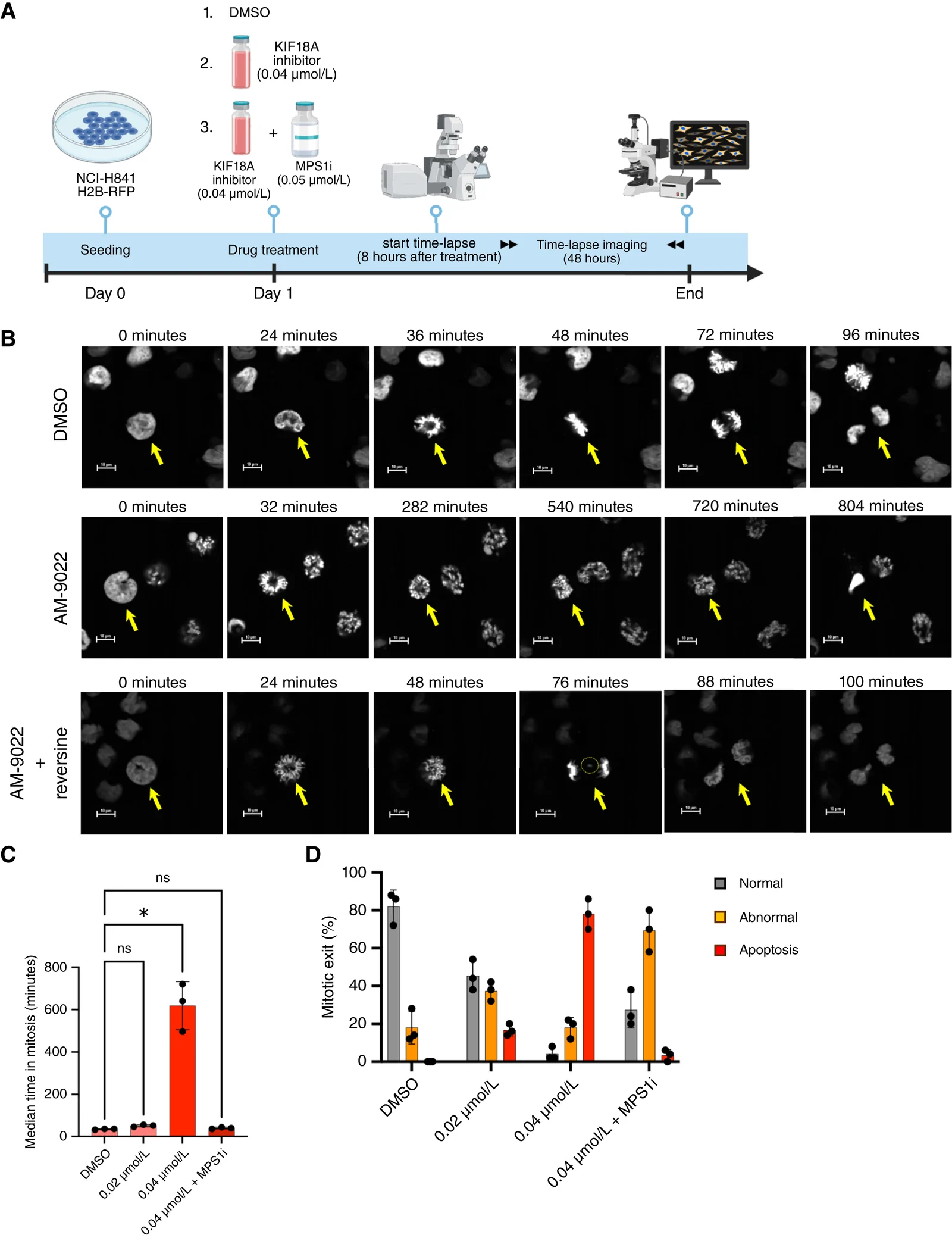
SAC-dependent mitotic arrest and cell fate outcomes following KIF18A inhibition in sensitive SCLC cells. **A,** Schematic timeline illustrating the experimental workflow for time-lapse imaging. MPS1i, MPS1 inhibitor. **B,** Representative time-lapse images of H2B-RFP–expressing NCI-H841 cells treated with DMSO, AM-9022 (0.04 μmol/L), or AM-9022 in combination with the SAC inhibitor reversine (0.05 μmol/L) showing mitotic outcome. Yellow arrows indicate mitotic cells. Scale bars, 10 μm. **C,** Quantification of mitotic timing in NCI-H841 cells following the indicated treatments. Mitotic timing was calculated by counting frames, each frame equivalent to 4 minutes, from nuclear envelope breakdown to anaphase onset or cell death in time-lapse images. Bars represent the median, and dots indicate individual biological replicates (*n* = 3). *P* values were calculated using the Friedman test with the Dunn multiple-comparison test. ns, not statistically significant; *, *P* < 0.05. **D,** Quantification of cell fate outcomes in NCI-H841 cells following the indicated treatments. Bar graphs show the percentage of cells undergoing normal or abnormal mitotic exit or apoptosis. Dots indicate individual biological replicates (*n* = 3). In **C** and **D,** 0.02 and 0.04 μmol/L denote the concentrations of AM-9022, and MPS1i denotes 0.05 μmol/L reversine, an MPS1 inhibitor.

Having established that KIF18A is required for proper metaphase chromosome alignment and mitotic progression in AM-9022–sensitive cell lines, we next used time-lapse imaging to examine how KIF18A inhibition affects mitotic progression and cell fate in these cells. H841 cells stably expressing RFP-tagged histone H2B (H841-H2B-RFP) were treated with AM-9022 for 8 hours prior to imaging, followed by continuous time-lapse acquisition for 48 hours (Fig. 4A). Fluorescently tagged H2B was used as a nuclear marker to assess chromosome dynamics and segregation fidelity.

Representative time-lapse images illustrate mitotic timing from nuclear envelope breakdown until either entry into anaphase or cell death across the indicated treatment conditions (Fig. 4B). Upon treatment with AM-9022, cells exhibited prolonged mitotic arrest, ultimately culminating in apoptotic cell death. Quantitative analysis demonstrated that AM-9022 induced a dose-dependent mitotic delay in H841-H2B-RFP cells (Fig. 4C), followed by either mitotic slippage with aberrant chromosome segregation or cell death, predominating at higher drug concentrations (Fig. 4D; Supplementary Fig. S4). In contrast, most DMSO-treated cells progressed through mitosis normally.

To test whether this prolonged mitotic arrest was dependent on SAC signaling, H841-H2B-RFP cells were cotreated with a low dose of reversine (0.05 μmol/L), an MPS1 inhibitor, and AM-9022. Notably, cotreatment, which partially overrides SAC signaling, restored mitotic timing comparable with DMSO control (Fig. 4C). However, under these conditions, a substantial fraction of cells exited mitosis with abnormal chromosome segregation (Fig. 4D), indicating that partial SAC override permits mitotic exit despite persistent segregation defects in AM-9022–treated H841 cells.

Collectively, these findings indicate that KIF18A inhibition triggers sustained SAC activation, leading to prolonged mitotic arrest and cell death in AM-9022–sensitive SCLC cells. Pharmacologic attenuation of SAC signaling abrogates mitotic arrest, underscoring SAC activation as a key mediator of KIF18A inhibitor–induced lethality.

### Reduced MAD1/BUBR1 recruitment and impaired SAC activation in resistant cells

We next investigated whether the defects in mitotic arrest upon treatment with AM-9022 in resistant DMS 53 cells reflect intrinsic defects in SAC signaling. To investigate this, DMS 53 and H841 cells were treated with monastrol, an KIF11 (EG5: kinesin-5) inhibitor to enrich the population prometaphase cells, a point at which SAC activation peaks because of the presence of tensionless kinetochores (38).

**Figure 5.**
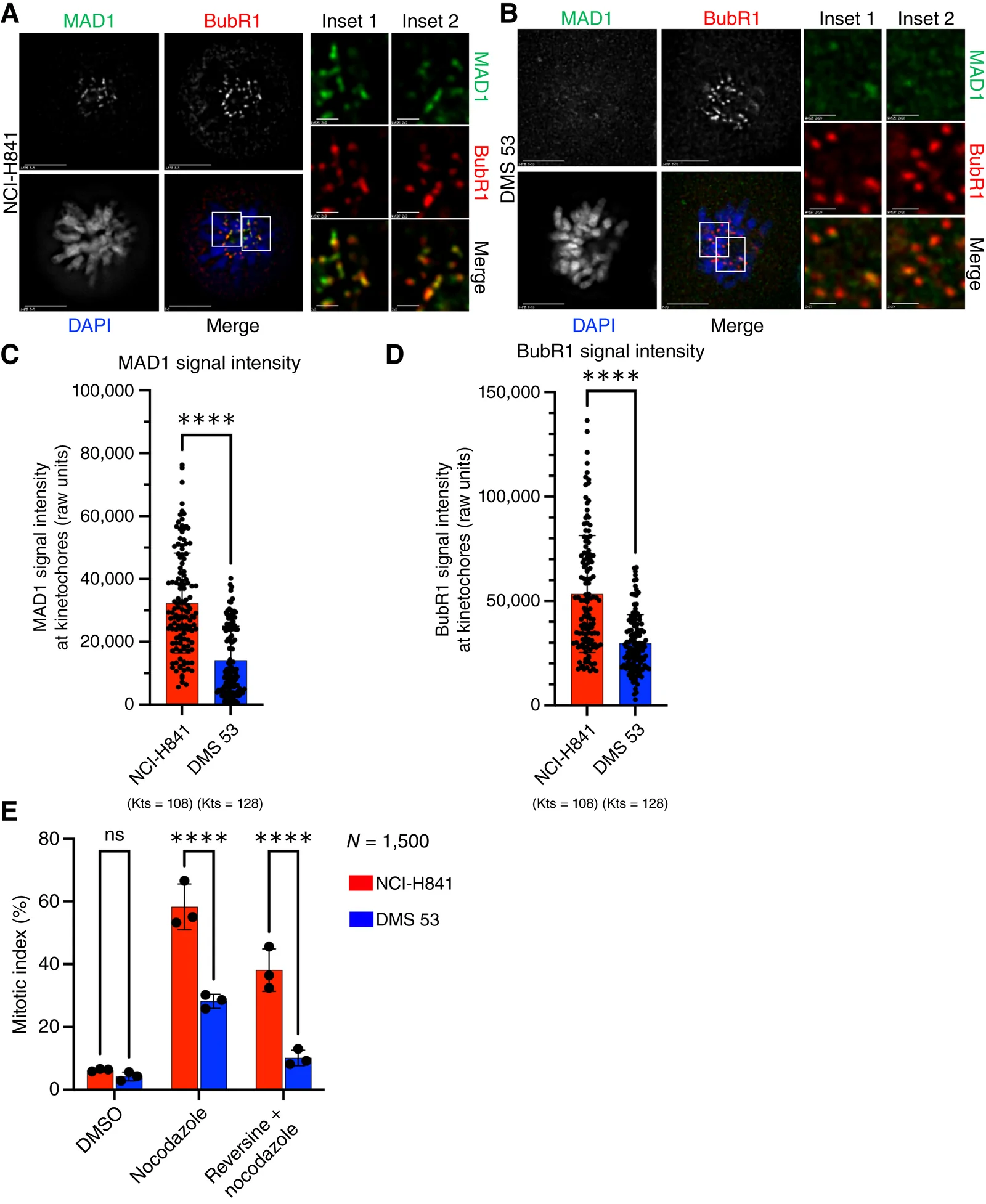
Differential SAC signaling at kinetochores in KIF18A inhibitor–sensitive and –resistant SCLC cells. **A,** Representative IF images showing localization of MAD1 (green) and BUBR1 (red) at prometaphase kinetochores in monastrol-treated NCI-H841 cells. Insets, zoomed images from white boxed areas in main images. Scale bars, 5 μm for main images and 1 μm for insets. **B,** Representative IF images showing localization of MAD1 (green) and BUBR1 (red) at prometaphase kinetochores in monastrol-treated DMS 53 cells. Insets, zoomed images from white boxed areas in main images. Scale bars, 5 μm for main images and 1 μm for insets. **C,** Quantification of prometaphase kinetochore-associated MAD1 signal intensities in monastrol-treated NCI-H841 and DMS 53 cells. Bar represents mean ± SD across kinetochores (*n* = 108 for H841 and *n* = 128 for DMS 53). Kinetochores from at least 12 cells from two independent experiments were analyzed. Each dot represents a single kinetochore. **D,** Quantification of prometaphase kinetochore-associated BUBR1 signal intensities in monastrol-treated NCI-H841 and DMS 53 cells. Bar represents mean ± SD across kinetochores (*n* = 108 for H841 and *n* = 128 for DMS 53). Kinetochores from at least 12 cells from two independent experiments were analyzed. Each dot represents a single kinetochore. **E,** Quantification of the mitotic index following nocodazole treatment in NCI-H841 and DMS 53 cells. Cells were treated with DMSO, nocodazole, or nocodazole in combination with the SAC inhibitor reversine. A total of 1,500 cells were counted for each cell line and for each condition across three biological replicates. Dots indicate individual biological replicates (*n* = 3). Data are presented as mean ± SD. *P* values were calculated using two-way ANOVA with cell line and treatment as factors. *P* values in **C** and **D** were calculated using the Welch test. ns, not statistically significant; ****, *P* < 0.0001. Kts, kinetochores.

Cells were immunostained with MAD1 and BUBR1, core components of the mitotic checkpoint complex, followed by localization and quantitative analysis of MAD1 and BUBR1 at prometaphase kinetochores. As expected for SAC-active state, MAD1 and BUBR1 localizations at kinetochores were intact in H841 cells, indicating intact SAC signaling. In contrast, DMS 53 cells exhibited reduced recruitment of MAD1 and BUBR1 at kinetochores, suggesting compromised SAC function (Fig. 5A and B). Quantitative IF analysis confirmed that the signal intensities of MAD1 and BUBR1 at prometaphase kinetochores were significantly reduced in DMS 53 cells compared with H841 cells (Fig. 5C and D), demonstrating a deficient SAC signaling in resistant cells. These findings were reproducible across additional SCLC models, with similar results observed in H524A (sensitive) and DMS 114 (resistant) cell lines (Supplementary Fig. S5A–S5D). Importantly, MAD1 signal intensities at the nuclear membrane in DMS 53 and H841 cells were comparable (Supplementary Fig. S5E and S5F), confirming that not overall but only the kinetochore recruitment of MAD1 was affected in resistant cells.

To functionally validate these observations, we challenged cells with a low dosage of nocodazole to induce microtubule depolymerization. In SAC-proficient cells, nocodazole treatment enforces mitotic arrest, whereas SAC-deficient cells undergo premature mitotic exit. Consistent with intact checkpoint function, H841 cells displayed a pronounced mitotic arrest. In contrast, DMS 53 cells failed to sustain mitotic arrest under the same conditions (Fig. 5E). Notably, DMS 53 cells treated with nocodazole alone exited mitosis prematurely than H841 cells cotreated with nocodazole and reversine, indicating that SAC in DMS 53 cells is intrinsically inefficient, even relative to pharmacologically induced SAC-deficient H841 cells. Together, these data demonstrate that DMS 53 cells are functionally deficient in SAC activation, both at the level of kinetochore signaling and checkpoint response, providing a mechanistic explanation for their resistance to KIF18A inhibition–induced mitotic arrest. Collectively, these results establish impaired SAC signaling as a reproducible feature of KIF18A inhibitor–resistant SCLC cell lines.

### Induction of CIN partially sensitizes resistant SCLC cells to KIF18A inhibition

Although resistant cells exhibit attenuated SAC signaling (Fig. 5), we reasoned that further increasing CIN might exceed the tolerance threshold and expose a dependence on mitotic fidelity mechanisms, including KIF18A. We therefore pretreated DMS 53 cells with a low dose of reversine to induce CIN and evaluated whether this manipulation alters their response to KIF18A inhibition. We first confirmed that 24 hours of treatment with a low dose of reversine was sufficient to induce atypical nuclei, a feature of CIN and aneuploidy, in DMS 53 cells (Supplementary Fig. S6A). Additionally, we confirmed that cell viability and cell proliferation were not significantly affected upon treatment with reversine for 24 hours prior to treatment with AM-9022 (Supplementary Fig. S6B). Notably, after 72 hours of cotreatment, cell viability and proliferation both were significantly reduced compared with treatment with AM-9022 or reversine alone (Supplementary Fig. S6B).

**Figure 6.**
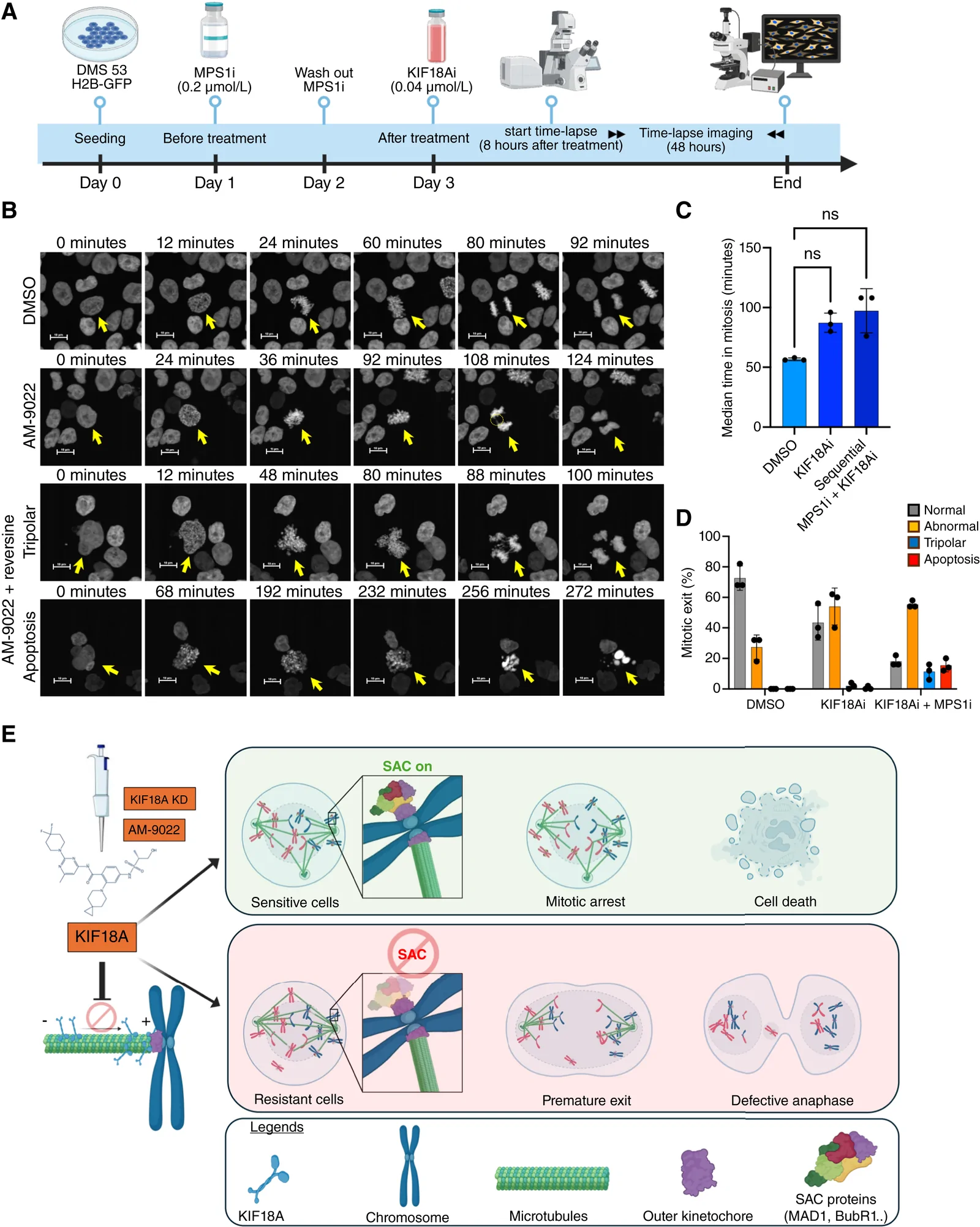
SAC integrity determines cellular responses to KIF18A inhibition in SCLC. **A,** Schematic timeline illustrating the experimental workflow for time-lapse imaging. KIF18Ai, KIF18A inhibitor; MPS1i, MPS1 inhibitor. **B,** Representative time-lapse images of H2B-GFP–expressing DMS 53 cells treated with DMSO, AM-9022 (0.04 μmol/L), or AM-9022 following pretreatment with the SAC inhibitor reversine. Representative frames from the time-lapse sequences. Yellow arrows indicate mitotic cells. Scale bars, 10 μm. **C,** Quantification of mitotic timing in DMS 53 cells following the indicated treatments. Mitotic timing was calculated by counting frames, each frame equivalent to 4 minutes, from nuclear envelope breakdown to anaphase onset or cell death in time-lapse images. Bars represent the median, and dots indicate individual biological replicates (*n* = 3). *P* values were calculated using the Friedman test with the Dunn multiple-comparison test. ns, not statistically significant. **D,** Quantification of cell fate outcomes in DMS 53 cells following the indicated treatments. Bar graphs show the percentage of cells undergoing normal or abnormal mitotic exit, tripolar, or apoptosis. Dots represent individual biological replicates (*n* = 3). **E,** Proposed model depicting how SAC integrity shapes cellular responses to KIF18A inhibition in SCLC. In AM-9022–sensitive cells, KIF18A inhibition causes chromosome misalignment, prolonged SAC activation, mitotic arrest, and cell death. In contrast, resistant cells exhibit attenuated SAC signaling and are able to progress through mitosis despite KIF18A inhibition, allowing continued survival and proliferation. KD, knockdown.

We then employed a sequential treatment strategy in DMS 53 cells (Fig. 6A). Cells were first pretreated with reversine for 24 hours to induce CIN. Following drug washout and recovery for 24 hours, cells were subsequently treated with AM-9022, and long-term live-cell time-lapse imaging was performed.

Representative time-lapse images illustrate mitotic behavior under this sequential treatment condition (Fig. 6B). In the resistant DMS 53 cell line, treatment with AM-9022 alone did not result in a significant prolongation of mitotic timing, and sequential pretreatment with reversine led to only a modest, nonsignificant extension of mitosis (Fig. 6C). However, quantitative analyses revealed that a subset of DMS 53 cells underwent cell death following reversine pretreatment and subsequent AM-9022 treatment, whereas AM-9022 treatment alone failed to induce detectable cell death in this model (Fig. 6D; Supplementary Fig. S6C). Together, these results indicate that induction of CIN partially resensitizes resistant SCLC cells to KIF18A inhibition, supporting a checkpoint-dependent, inducible vulnerability to mitotic perturbation.

## Discussion

SCLC is one of the most chromosomally unstable human malignancies, characterized by pervasive aneuploidy, high replication stress, and rapid proliferative turnover (3, 27). Such extreme CIN has been proposed to generate targetable mitotic vulnerabilities (22). Among these, the mitotic kinesin KIF18A, a kinesin 8 family motor that suppresses microtubule plus-end dynamics and promotes chromosome alignment, has emerged as a selective dependency in CIN-high tumors in multiple cancer models (15, 22, 24, 25). More recently, pharmacologic KIF18A inhibition with VLS-1272 was also shown to induce chromosome congression defects, mitotic accumulation, and cell death preferentially in CIN-high models (23). In this study, we investigated whether this CIN-associated dependency extends to SCLC, a prototypical CIN-high cancer.

Among lung cancer histologic subtypes, *KIF18A* expression was particularly elevated in SCLC and strongly correlated with CIN-associated gene signatures such as CES (28), CIN70 (29), and proliferation markers (MKI67 and PCNA). These findings indicate that KIF18A expression tracks with the high mitotic activity and genomic instability that characterize SCLC, particularly NE models (17). However, KIF18A abundance did not predict therapeutic response; across our SCLC cell line panel, neither KIF18A mRNA nor KIF18A protein levels correlated with AM-9022 sensitivity, and response did not segregate by transcription factor–defined subtype.

This is consistent with prior studies showing that KIF18A dependency is shaped not by target expression alone but by the broader mitotic state of the cell, including CIN, aneuploidy, spindle dynamics, SAC output, and APC/C activity (15, 22–24). Thus, our study does not identify *KIF18A* mRNA or KIF18A protein expression as a predictive biomarker in SCLC. Instead, it extends prior observations to the SCLC context and suggests that functional checkpoint competence may contribute to response heterogeneity among SCLC models. Consistent with earlier work implicating SAC signaling and SAC/APC/C balance in KIF18A inhibitor response, our data provide SCLC-specific evidence that this axis is operative in this disease.

Transcriptomic comparisons of AM-9022–sensitive and –resistant cell lines revealed distinct programs related to mitotic spindle organization, checkpoint regulation, and cell-cycle control. Sensitive lines showed enrichment of CIN-associated and spindle assembly gene sets, including higher CESs, suggesting a cellular state with greater reliance on mitotic checkpoint function rather than simply faster proliferation. Consistent with this, proliferation rate analysis in the available cell line subset did not show a clear association with AM-9022 sensitivity (Supplementary Fig. S2E).

EMT-related gene sets were also enriched in the sensitive group, whereas MYC target programs were relatively enriched in resistant lines. Although EMT and MYC-family programs can influence therapeutic response in other contexts, the present study was not designed to define their functional contribution to KIF18A inhibitor sensitivity. Given the limited size and biological heterogeneity of the cell line panel, including variability in *TP53/RB1* and MYC-family alteration status (Supplementary Table S4), these pathway-level associations should be interpreted as exploratory and hypothesis generating rather than as independent predictors of AM-9022 response.

Functionally, resistant lines exhibited reduced expression and kinetochore localization of core SAC components MAD1 and BUBR1, consistent with attenuated checkpoint signaling. These findings resemble SAC-compromised models, in which cells prematurely exit mitosis despite spindle perturbation (19), and are consistent with prior KIF18A inhibitor studies showing that reduced SAC signaling, including through impaired MAD1/CCNB1 (cyclin B1) function or pharmacologic MPS1 inhibition, can limit KIF18A inhibitor toxicity by permitting mitotic exit or slippage despite chromosome alignment defects (21, 23).

Accordingly, live-cell imaging demonstrated that KIF18A inhibition triggered pronounced mitotic arrest and apoptosis in SAC-proficient cells, whereas SAC-deficient cells underwent only mild mitotic delay and survived despite chromosome segregation errors. Together, these findings support a potential role for SAC competence in shaping vulnerability to KIF18A inhibition in SCLC.

Mechanistically, these results support a model in which SAC-proficient SCLC cells rely on KIF18A to maintain spindle tension and alignment fidelity under conditions of CIN. When KIF18A is inhibited, persistent kinetochore–microtubule attachment defects activate SAC signaling and trigger apoptotic death, whereas SAC-deficient cells fail to sustain arrest and instead proceed through erroneous mitosis, preserving viability (Fig. 6E). This model is aligned with prior work showing that KIF18A inhibition causes chromosome congression defects, mitotic checkpoint activation, mitotic accumulation, and cell death in CIN-high tumor models (15, 23, 24). Our contribution is to show that a similar response pattern is observed in SCLC and that resistant SCLC models are characterized by attenuated induction or maintenance of SAC markers. This is consistent with recent work showing that SAC defects confer tolerance to mitotic stressors such as spindle poisons (39, 40), as well as with KIF18A-specific studies indicating that reduced SAC signaling or altered SAC/APC/C balance can blunt the cytotoxic consequences of KIF18A inhibition (21, 23).

From a translational perspective, SAC integrity may serve as a candidate functional biomarker for predicting response to KIF18A-targeted therapy. Given the heterogeneous SAC status across SCLC models, therapeutic combinations that modulate checkpoint signaling could enhance efficacy. However, such combinations require careful consideration of treatment schedule and mechanism. Prior studies indicate that acute attenuation of SAC signaling, for example, through MPS1 inhibition or partial SAC suppression, can reduce KIF18A inhibitor toxicity by permitting mitotic exit (21, 23). Therefore, our sequential reversine–AM-9022 strategy should not be interpreted simply as SAC inhibition sensitizing cells to KIF18A inhibition. Rather, our data support a schedule-dependent “CIN-escalation” model in which transient checkpoint perturbation may increase chromosome segregation errors and push resistant SCLC cells beyond their tolerance threshold before KIF18A inhibition is applied. Indeed, our data support a synthetic lethal framework in which transient amplification of CIN, achieved by sequential reversine treatment followed by AM-9022, can resensitize resistant cells by exceeding their tolerance for mitotic errors. Such “CIN-escalation” approaches may extend the therapeutic reach of KIF18A inhibition beyond SAC-proficient tumors, aligning with emerging concepts that controlled CIN elevation can potentiate mitotic stress therapies (41). Nevertheless, because SAC suppression can also antagonize KIF18A inhibitor–induced mitotic arrest, the therapeutic window, dose, and sequence of this strategy will require rigorous validation in additional SCLC models and *in vivo* systems.

Several limitations should be acknowledged. First, the number of cell lines analyzed, although representative, remains limited for capturing the full molecular diversity of SCLC and for determining whether oncogenic features, including MYC-family status or p53-associated pathway activity, independently predict AM-9022 response. Second, mechanistic findings were derived from *in vitro* models; *in vivo* validation using patient-derived xenograft or organoid systems will be essential to confirm SAC proficiency as a predictive biomarker. Third, AM-9022 remains a preclinical compound with incompletely defined pharmacologic and off-target profiles. Finally, although SAC function emerged as the dominant determinant of response in our SCLC cell line panel, emerging evidence suggests that other mitotic regulators or metabolic adaptations may also modulate KIF18A dependency (42). In addition, prior KIF18A inhibitor studies have highlighted multiple nonmutually exclusive determinants of response, including CIN level, aneuploidy, kinetochore–microtubule attachment stability, SAC output, and APC/C activity (15, 21–25). Future work integrating proteogenomic and phospho-signaling analyses in clinical SCLC samples will be crucial to refine predictive models for KIF18A-targeted therapy.

In summary, this study extends the previously described association of CIN, SAC signaling, and KIF18A dependency with SCLC. KIF18A inhibition preferentially induces mitotic arrest and apoptosis in SAC-proficient, CIN-high cells, whereas SAC-deficient cells bypass arrest and continue proliferating despite aberrant segregation (Fig. 6E). The novelty of this study is therefore not the initial identification of SAC deficiency as a mechanism of resistance to KIF18A inhibition, but the demonstration that this mechanism is relevant in SCLC and that the *KIF18A* mRNA or KIF18A protein expression level or transcription factor–defined SCLC subtype alone is insufficient to predict response. These findings position SAC integrity as a candidate biomarker and potential therapeutic lever in SCLC and provide a rational basis for KIF18A-directed strategies in genomically unstable yet checkpoint-competent tumors.

## Supplementary Material

Supplementary Figure S1 Figure S1. Sensitivity analyses evaluating dataset-dependent effects in cross-cohort RNA expression comparisons and KIF18A-associated features in SCLC datasets.

Supplementary Figure S2 Figure S2. Supplementary analyses supporting the stratification of SCLC cell lines by sensitivity to KIF18A inhibition.

Supplementary Figure S3 Figure S3. Mitotic index following pharmacologic KIF18A inhibition and validation of doxycycline-inducible KIF18A knockdown in SCLC cells.

Supplementary Figure S4 Figure S4. Single-cell analysis of mitotic timing and fate following KIF18A inhibition in NCI-H841 cells.

Supplementary Figure S5 Figure S5. Extended analysis of kinetochore and nuclear envelope localization of spindle assembly checkpoint components in SCLC cells.

Supplementary Figure S6 Figure S6. Effects of reversine and sequential AM-9022 treatment in AM-9022-resistant SCLC cells and single-cell analysis of mitotic timing and fate in DMS53 cells following KIF18A inhibition.

Supplementary Table S1 Table S1. Sequences of shRNAs targeting KIF18A used in this study.

Supplementary Table S2 Table S2. KIF18A expression, CES, RepStress score, and NE score were calculated from the RNA-sequencing dataset indicated for each cell line, including either in-house RNA-seq data or publicly available RNA-seq data. KIF18A expression is shown as log2-transformed TPM values.

Supplementary Table S3 Table S3. Doubling time is expressed in days.

Supplementary Table S4 Table S4. Summary of reported alterations in major SCLC-relevant tumor suppressor and oncogenic drivers, including TP53, RB1, MYC, MYCN, and MYCL, for the cell lines analyzed in this study.

## Data Availability

The RNA-seq data generated from the patient-derived cell lines are available on the Database of Genotypes and Phenotypes (dbGaP) under study accession phs004869.v1.p1. These data are available through dbGaP under controlled-access procedures, subject to applicable informed-consent and data-use limitations. All other data supporting the findings of this study are available from the corresponding author upon reasonable request.
