# Supplementary material for: Spindle Assembly Checkpoint Competency Determines Sensitivity to KIF18A Inhibition in Small Cell Lung Cancer
Source: Cancer Res Commun. 2026 Sep 9;6(9):2110–25. doi: 10.1158/2767-9764.CRC-26-0560 (PMC13555252; doi:10.1158/2767-9764.CRC-26-0560)
Supplement: Supplementary file 1 [file crc-26-0560_supplementary_figure_s1_suppsf1.docx]

**Supplementary Figure S1. Sensitivity analyses evaluating dataset-dependent effects in cross-cohort RNA expression comparisons and KIF18A-associated features in SCLC datasets.**

**A,** KIF18A mRNA expression across TCGA LUAD, TCGA LUSC, the George et al. SCLC cohort, and independent SCLC cohorts, including the Liu et al., Barzin, and Sehoon Park datasets.

**B,** CES scores across the same cohorts shown in A.

**C,** Expression levels of individual housekeeping genes, including ACTB, B2M, GAPDH, HPRT1, and RPLP0, in TCGA LUAD, TCGA LUSC, and the George et al. SCLC cohort.

**D,** Mean housekeeping gene expression across TCGA LUAD, TCGA LUSC, and the George et al. SCLC cohort.

**E,** KIF18A expression after adjustment for mean housekeeping gene expression in TCGA LUAD, TCGA LUSC, and the George et al. SCLC cohort. Adjusted values represent residual KIF18A expression after regressing KIF18A expression on mean housekeeping gene expression.

**F,** Correlations between KIF18A expression and CIN- or proliferation-associated features, including CES, CIN70, MKI67, and PCNA, in independent SCLC cohorts. Plots are shown for features available in each dataset. Samples were stratified into KIF18A-high and KIF18A-low groups based on cohort-specific KIF18A expression for visualization. Correlation coefficients and P values are indicated in each plot.

**G, H,** CES score (G) and MKI67 expression (H) across SCLC molecular subtypes in the George et al. cohort.
