# Supplementary material for: Spindle Assembly Checkpoint Competency Determines Sensitivity to KIF18A Inhibition in Small Cell Lung Cancer
Source: Cancer Res Commun. 2026 Sep 9;6(9):2110–25. doi: 10.1158/2767-9764.CRC-26-0560 (PMC13555252; doi:10.1158/2767-9764.CRC-26-0560)
Supplement: Supplementary file 2 [file crc-26-0560_supplementary_figure_s2_suppsf2.docx]

**Supplementary Figure S2.** **Supplementary analyses supporting the stratification of SCLC cell lines by sensitivity to KIF18A inhibition.**

**A,** Individual ATP-based dose–response curves for the 25 SCLC cell lines shown in Fig. 2D. For each cell line, two independent experiments are shown, each performed in triplicate, resulting in two dose–response curves per cell line. Cell lines were grouped by drug response at the highest tested concentration: sensitive if viability was <50%, resistant if viability was ≥85% (Imax_obs <15%), and partial/excluded if viability remained ≥50% despite measurable inhibition (Imax_obs ≥10–15%).

**B,** Correlation analysis showing the relationship between normalized area under the curve (AUC) values for AM-9022 sensitivity and KIF18A mRNA expression across a panel of 25 SCLC cell lines. No significant correlation was observed.

**C,** Correlation analysis showing the relationship between normalized AUC values and neuroendocrine (NE) scores across 25 SCLC cell lines. No significant correlation was observed.

**D,** Correlation analysis showing the relationship between normalized AUC values and KIF18A protein expression across 15 SCLC cell lines. No significant correlation was observed.

**E,** Correlation analysis showing the relationship between cell doubling times and AM-9022 normalized AUC values across six SCLC cell lines. No significant correlation was observed, indicating that KIF18A inhibitor sensitivity is independent of cell proliferation rate.

**F,** GOBP enrichment analysis comparing AM-9022–sensitive and –resistant SCLC cell lines.
