# Supplementary material for: Spindle Assembly Checkpoint Competency Determines Sensitivity to KIF18A Inhibition in Small Cell Lung Cancer
Source: Cancer Res Commun. 2026 Sep 9;6(9):2110–25. doi: 10.1158/2767-9764.CRC-26-0560 (PMC13555252; doi:10.1158/2767-9764.CRC-26-0560)
Supplement: Supplementary file 3 [file crc-26-0560_supplementary_figure_s3_suppsf3.docx]

**Supplementary Figure S3.** **Mitotic index following pharmacologic KIF18A inhibition and validation of doxycycline-inducible KIF18A knockdown in SCLC cells.**

**A,** Cell viability measured by MTS assay following AM-9022 treatment in NCI-H841 and DMS53 cells. H841 cells show a marked reduction in viability, whereas DMS53 cells remain relatively resistant. IC_50_ values derived from dose–response curves are indicated (H841 ≈ 0.032 μM; DMS53 > 10 μM).

**B,** Quantification of mitotic index in H841, H524A, DMS53, and DMS114 cells. Cells were treated with DMSO or AM-9022. 1500 cells were counted for each cell line and for each condition across three biological replicates. Dots indicate individual biological replicates (n = 3). Data are presented as mean ± SD. P values were calculated using one-way ANOVA with Tukey’s test. ****, P < 0.0001.

**C,** Immunoblot analysis showing KIF18A protein levels in whole cell extracts of NCI-H841 cells treated with different concentrations of DOX to induce KIF18A shRNA_1. α-Tubulin was used as a loading control.

**D,** Immunoblot analysis showing KIF18A protein levels in whole cell extracts of DMS53 treated with different concentrations of DOX to induce KIF18A shRNA_2. α-Tubulin was used as a loading control.
