# Supplementary material for: Spindle Assembly Checkpoint Competency Determines Sensitivity to KIF18A Inhibition in Small Cell Lung Cancer
Source: Cancer Res Commun. 2026 Sep 9;6(9):2110–25. doi: 10.1158/2767-9764.CRC-26-0560 (PMC13555252; doi:10.1158/2767-9764.CRC-26-0560)
Supplement: Supplementary file 4 [file crc-26-0560_supplementary_figure_s4_suppsf4.docx]

**Supplementary Figure S4.** **Single-cell analysis of mitotic timing and fate following KIF18A inhibition in NCI-H841 cells.**

Quantification of mitotic timing in NCI-H841 cells under the indicated treatment conditions. Each dot represents a single cell measured by time-lapse imaging from nuclear envelope breakdown to either anaphase onset or cell death. For each condition, 50 mitotic cells were analyzed and classified based on mitotic outcome as normal mitotic exit, abnormal mitotic exit, or apoptosis.
