# Supplementary material for: Spindle Assembly Checkpoint Competency Determines Sensitivity to KIF18A Inhibition in Small Cell Lung Cancer
Source: Cancer Res Commun. 2026 Sep 9;6(9):2110–25. doi: 10.1158/2767-9764.CRC-26-0560 (PMC13555252; doi:10.1158/2767-9764.CRC-26-0560)
Supplement: Supplementary file 5 [file crc-26-0560_supplementary_figure_s5_suppsf5.docx]

**Supplementary Figure S5.** **Extended analysis of kinetochore and nuclear envelope localization of spindle assembly checkpoint components in SCLC cells.**

**A,** Representative immunofluorescence images showing localization of MAD1 (green) and BubR1 (red) at prometaphase kinetochores in monastrol treated NCI-H524A cells. Insets show zoomed images from white boxed areas in main images. Scale bar: 5 μm for main images and 1 μm for insets.

**B,** Representative immunofluorescence images showing localization MAD1 (green) and BubR1 (red) at prometaphase kinetochores in monastrol treated DMS114 cells. Insets show zoomed images from white boxed areas in main images. Scale bar: 5 μm for main images and 1 μm for insets.

**C,** Quantification of prometaphase kinetochore-associated MAD1 signal intensities in monastrol treated NCI-H524A and DMS114 cells. Bar represents mean ± SD across kinetochores (n=72). Kinetochores from at least 6 cells from two independent experiments were analyzed. Each dot represents a single kinetochore.

**D,** Quantification of prometaphase kinetochore-associated BubR1 signal intensities in NCI-H524A and DMS114 cells. Bar represents mean ± SD across kinetochores (n=72). Kinetochores from at least 6 cells from two independent experiments were analyzed. Each dot represents a single kinetochore.

**E,** Representative projected immunofluorescence images showing localization of nuclear membrane associated MAD1 in interphase NCI-H841 and DMS53 cells.

**F,** Quantification of nuclear membrane-associated MAD1 signal intensities in NCI-H841 and DMS53 cells. Bar represents mean ± SD across average cellular intensity in at least 50 cells from two independent experiments. Each dot represents an average intensity of MAD1 in each cell.

P values in panels C, D and F were calculated using Welch’s test.

****, P < 0.0001.
