# Supplementary material for: Spindle Assembly Checkpoint Competency Determines Sensitivity to KIF18A Inhibition in Small Cell Lung Cancer
Source: Cancer Res Commun. 2026 Sep 9;6(9):2110–25. doi: 10.1158/2767-9764.CRC-26-0560 (PMC13555252; doi:10.1158/2767-9764.CRC-26-0560)
Supplement: Supplementary file 6 [file crc-26-0560_supplementary_figure_s6_suppsf6.docx]

**Supplementary Figure S6. Effects of reversine and sequential AM-9022 treatment in AM-9022-resistant SCLC cells and single-cell analysis of mitotic timing and fate in DMS53 cells following KIF18A inhibition.**

**A,** Reversine-induced atypical nuclei in a resistant cell line. Nuclear morphology was assessed in an AM-9022-resistant SCLC cell line following reversine treatment.

**B,** Cell viability and proliferation following sequential reversine and AM-9022 treatment. AM-9022-resistant SCLC cells were pretreated with reversine followed by AM-9022 treatment. Cell viability and proliferation were assessed to evaluate the effect of sequential treatment.

**C,** Quantification of mitotic timing in DMS53 cells under the indicated treatment conditions. Each dot represents a single cell measured by time-lapse imaging from nuclear envelope breakdown to either anaphase onset or cell death. For each condition, 50 mitotic cells were analyzed and classified based on mitotic outcome as normal mitotic exit, abnormal mitotic exit, tripolar, or apoptosis.
