# Supplementary material for: Spindle Assembly Checkpoint Competency Determines Sensitivity to KIF18A Inhibition in Small Cell Lung Cancer
Source: Cancer Res Commun. 2026 Sep 9;6(9):2110–25. doi: 10.1158/2767-9764.CRC-26-0560 (PMC13555252; doi:10.1158/2767-9764.CRC-26-0560)
Supplement: Supplementary file 7 [file crc-26-0560_supplementary_table_s1_suppst1.docx]

| shRNA | Seq 5’ to 3’ |
| --- | --- |
| shKIF18A_1F | ACCGGATTCTACGATGACACATATAAGTTAATATTCATAGCTTATATGTGTCATCGTAGAATTTTT |
| shKIF18A_1R | CGAAAAAAATTCTACGATGACACATATAAGCTATGAATATTAACTTATATGTGTCATCGTAGAATC |
| shKIF18A_2F | ACCGGGTCGTTCATGGACTTACTTTAGTTAATATTCATAGCTAAAGTAAGTCCATGAACGACTTTT |
| shKIF18A_2R | CGAAAAAAGTCGTTCATGGACTTACTTTAGCTATGAATATTAACTAAAGTAAGTCCATGAACGACC |

**Supplementary Table S1.** Sequences of shRNAs targeting KIF18A used in this study.
