# Supplementary material for: Spindle Assembly Checkpoint Competency Determines Sensitivity to KIF18A Inhibition in Small Cell Lung Cancer
Source: Cancer Res Commun. 2026 Sep 9;6(9):2110–25. doi: 10.1158/2767-9764.CRC-26-0560 (PMC13555252; doi:10.1158/2767-9764.CRC-26-0560)
Supplement: Supplementary file 8 [file crc-26-0560_supplementary_table_s2_suppst2.docx]

| Cell line | KIF18A expression (log2 TPM) | CES score | Repstress score | NE score | NE status | Subtype |
| --- | --- | --- | --- | --- | --- | --- |
| NCI-H841 | 3.20 | 36.4 | 0.73 | -0.25 | NonNE | YAP1 |
| NCI-H446_S | 3.12 | 35.0 | 0.81 | 0.80 | NE | NEUROD1 |
| NCI-H524_A | 3.02 | 35.0 | 0.71 | 0.14 | NE | ASCL1 |
| NCI-H526 | 3.39 | 34.7 | 0.75 | 0.38 | NE | POU2F3 |
| NCI-H82 | 2.94 | 34.7 | 0.79 | 0.57 | NE | NEUROD1 |
| DMS 53 | 2.36 | 29.4 | 0.59 | 0.29 | NE | ASCL1 |
| DMS 114 | 2.95 | 35.2 | 0.71 | -0.08 | NonNE | YAP1 |
| COLO_668 | 2.89 | 32.7 | 0.67 | 0.60 | NE | ASCL1 |
| NCI-H1092 | 3.23 | 35.0 | 0.77 | 0.81 | NE | ASCL1 |
| NCI-H889 | 3.22 | 34.6 | 0.76 | 0.74 | NE | ASCL1 |
| COR_L88 | 3.44 | 35.1 | 0.85 | 0.66 | NE | ASCL1 |
| NCI-H1963 | 3.28 | 36.3 | 0.82 | 0.82 | NE | ASCL1 |
| NCI-H69 | 3.33 | 35.1 | 0.81 | 0.60 | NE | ASCL1 |
| NCI-H146 | 3.32 | 36.0 | 0.82 | 0.78 | NE | ASCL1 |
| SW_1271 | 2.46 | 32.9 | 0.51 | -0.67 | NonNE | YAP1 |
| NCI_H2171 | 3.32 | 35.7 | 0.79 | 0.69 | NE | NEUROD1 |
| NCI_H2029 | 2.99 | 32.4 | 0.64 | 0.62 | NE | ASCL1 |
| NCI_H1048 | 2.68 | 32.1 | 0.55 | -0.02 | NonNE | POU2F3 |
| NCI_H211 | 3.18 | 34.7 | 0.70 | -0.07 | NonNE | POU2F3 |
| NCI-H446_A | 3.02 | 34.2 | 0.70 | -0.02 | NonNE | YAP1 |
| NCI-H524_S | 2.82 | 33.5 | 0.75 | 0.91 | NE | NEUROD1 |
| DMS 273_A | 2.93 | 33.8 | 0.75 | 0.55 | NE | ASCL1 |
| DMS 273_S | 2.97 | 34.4 | 0.79 | 0.13 | NE | ASCL1 |
| RA22#12_A | 3.13 | 34.0 | 0.69 | -0.25 | NonNE | YAP1 |
| RA22#12_S | 3.01 | 35.2 | 0.79 | 0.95 | NE | ASCL1 |

**Supplementary Table S2.** KIF18A expression, CES, RepStress score, and NE score were calculated from the RNA-sequencing dataset indicated for each cell line, including either in-house RNA-seq data or publicly available RNA-seq data. KIF18A expression is shown as log2-transformed TPM values. CES was calculated using a previously defined centromere and kinetochore gene expression signature based on gene-level z-scores. RepStress and NE scores were calculated using previously described scoring methods. NE status and molecular subtype classification were assigned based on established thresholds and molecular criteria.
