# Supplementary material for: Spindle Assembly Checkpoint Competency Determines Sensitivity to KIF18A Inhibition in Small Cell Lung Cancer
Source: Cancer Res Commun. 2026 Sep 9;6(9):2110–25. doi: 10.1158/2767-9764.CRC-26-0560 (PMC13555252; doi:10.1158/2767-9764.CRC-26-0560)
Supplement: Supplementary file 9 [file crc-26-0560_supplementary_table_s3_suppst3.docx]

| Cell line | Doubling time  (day) | Proliferation rate (%/day) | AM-9022 Sensitivity |
| --- | --- | --- | --- |
| DMS114 | 2.15 | 37.86 | Resistant |
| COLO668 | 1.25 | 73.65 | Resistant |
| NCI-H446A | 1.43 | 62.29 | Resistant |
| RA22#12A | 2.09 | 39.23 | Resistant |
| NCI-H841 | 1.35 | 66.61 | Sensitive |
| DMS53 | 1.82 | 46.23 | Resistant |

**Supplementary Table S3.** Doubling time is expressed in days. Proliferation rate is expressed as the percentage increase in cell number per day (%/day).
