# Supplementary material for: Spindle Assembly Checkpoint Competency Determines Sensitivity to KIF18A Inhibition in Small Cell Lung Cancer
Source: Cancer Res Commun. 2026 Sep 9;6(9):2110–25. doi: 10.1158/2767-9764.CRC-26-0560 (PMC13555252; doi:10.1158/2767-9764.CRC-26-0560)
Supplement: Supplementary file 10 [file crc-26-0560_supplementary_table_s4_suppst4.docx]

| Cell line | TP53 status | RB1 status | MYC status | MYCN status | MYCL status |
| --- | --- | --- | --- | --- | --- |
| NCI-H841 | MUT + AMP | ND | Neutral | Neutral | Neutral |
| NCI-H526 | MUT | MUT | Neutral | AMP | AMP |
| NCI-H82 | MUT | MUT | AMP | Neutral | Neutral |
| DMS 53 | MUT | ND | Neutral | Neutral | Neutral |
| DMS 114 | MUT | ND | Neutral | Neutral | Neutral |
| COLO668 | MUT | MUT + AMP | Neutral | Neutral | AMP |
| NCI-H1092 | MUT | ND | Neutral | Neutral | AMP |
| NCI-H889 | MUT | ND | Neutral | Neutral | AMP |
| COR-L88 | MUT | MUT | Neutral | Neutral | AMP |
| NCI-H1963 | MUT | MUT + loss | Neutral | Neutral | AMP |
| NCI-H69 | MUT + loss | MUT | Neutral | AMP | Neutral |
| NCI-H146 | MUT | MUT | Neutral | Neutral | Neutral |
| SW1271 | MUT | ND | Neutral | Neutral | Neutral |
| NCI-H2171 | MUT | MUT | AMP | Neutral | Neutral |
| NCI-H2029 | MUT | MUT | Neutral | Neutral | AMP |
| NCI-H1048 | MUT | MUT + loss | Neutral | Neutral | Neutral |
| NCI-H211 | MUT + loss | ND | AMP | Neutral | Neutral |
| NCI-H446 | MUT | MUT | AMP | Neutral | Neutral |
| NCI-H524 | MUT + loss | MUT | AMP | Neutral | Neutral |
| DMS 273 | MUT | MUT | AMP | Neutral | AMP |

**Supplementary Table S4.** Summary of reported alterations in major SCLC-relevant tumor suppressor and oncogenic drivers, including TP53, RB1, MYC, MYCN, and MYCL, for the cell lines analyzed in this study.
